# Emerging Role of Ultrasound in Dysphagia Assessment and Intervention: A Narrative Review

**DOI:** 10.3389/fresc.2021.708102

**Published:** 2021-08-11

**Authors:** Ming-Yen Hsiao, Chueh-Hung Wu, Tyng-Guey Wang

**Affiliations:** ^1^Department of Physical Medicine and Rehabilitation, College of Medicine, National Taiwan University, Taipei, Taiwan; ^2^Department of Physical Medicine and Rehabilitation, National Taiwan University Hospital, Taipei, Taiwan; ^3^Department of Physical Medicine and Rehabilitation, National Taiwan University Hospital Hsin-chu Branch, Hsinchu, Taiwan

**Keywords:** ultrasonography, swallowing function, dysphagia, deglutition disorders, hyolaryngeal excursion, tongue

## Abstract

Ultrasonography has gained increasing attention as a non-invasive and radiation-free instrument for the assessment of swallowing function. In the past decades, an extensive repertoire of ultrasonographic techniques, such as, B-mode dynamic scanning, pixel analysis, M-mode, Doppler, 3D reconstruction, and sonoelastography, has been applied in the evaluation of oropharyngeal structures and movement. Yet, a universal consensus on the examination protocols and clinical implications remains to be established. This review aimed to provide a brief introduction of the application of ultrasound in dysphagia assessment and intervention, encompassing the ultrasonography of swallowing-related muscles, tongue movement, and hyolaryngeal excursion, as well as ultrasound-guided interventions in the management of dysphagia. In addition to non-invasiveness, ultrasonography, a portable, easy to use, and low-cost technique, could compliment videofluoroscopic swallowing study as a first-line screening and follow-up tool for the evaluation of swallowing function, although further study is warranted to provide quantitative diagnostic and prognostic values. Finally, ultrasonography aids in the precisely targeted injection of botulinum toxin in patients exhibiting oropharyngeal muscle spasticity.

## Introduction

Oropharyngeal dysphagia affects up to one-third of older adults (1) and 40% of institutionalized residents (2). Patients with neurological disorders, cardiopulmonary diseases, and gastrointestinal diseases and those who have undergone endotracheal intubation or tracheostomy are commonly affected (1, 3–6) Complications of dysphagia include aspiration pneumonia, malnutrition, and dehydration (7, 8). However, a formal screening of swallowing function effectively reduces the risks of pneumonia (9, 10). Moreover, standard instrumental swallowing examinations such as the videofluoroscopic swallowing study (VFSS) (11) and fiberoptic endoscopic evaluation of swallowing (FEES) (12) are often not accessible in community settings, long-term care facilities, or many hospitals. Also, there are several limitations even in the settings with the required instruments. Radiation exposure hinders the use of VFSS as a tool for screening or serial follow-ups, although it is considered the gold standard for assessing oropharyngeal dysphagia. FEES is suitable for bedside evaluation, but there is a white-out during the pharyngeal phase of swallowing. Also, the endoscopic tube may cause discomfort and affect normal swallowing physiology.

In this regard, ultrasonography (US) has attracted increasing attention as a potential tool for the evaluation of swallowing function, owing to its non-invasiveness, radiation-free characteristic, accessibility, and negligible influence on the normal swallowing process (13, 14). US provides excellent resolution in depicting orofacial and neck musculatures (15–19) and has been used to observe airway and vocal folds movement (18, 20–22). Many studies have applied US to evaluate tongue and hyolaryngeal movement (23–30). Observations of the cricopharyngeal muscle (CPm) as well as US-guided intervention has also been reported (31–33). However, there is significant variation in examination techniques and methodology, and the clinical diagnostic value of US in dysphagia management remains to be established. Therefore, in this narrative review, we aimed to provide abroad overview of various US applications in the evaluation of swallowing function, with a particular focus on its clinical implications with regard to diagnosis and management of oropharyngeal dysphagia based on our experience. The detailed examination techniques and the application of US in the evaluation of pediatric swallowing and articulation disorders are beyond the scope of this article.

## Literature Search

We conducted a literature search in PubMed, MEDLINE, and Scopus for all types of articles published until July 8, 2021, using broad search terms of “ultrasound OR sonography OR ultrasonography” AND “swallow OR deglutition OR dysphagia OR tongue OR hyoid OR larynx OR upper esophageal sphincter OR cricopharyngeal muscle.” Additional articles were identified by manually searching the references of previously identified publications. Articles not written in English were excluded. Selected works of literature regarding the application of US in assessing the swallowing function in both healthy and diseased populations are included. It was a non-systemic search with a special focus on tongue and hyolaryngeal movement, submental muscle contraction, upper esophageal sphincter, and airway structures, as described below.

## Assessing Tongue Movement

**Figure 1:**
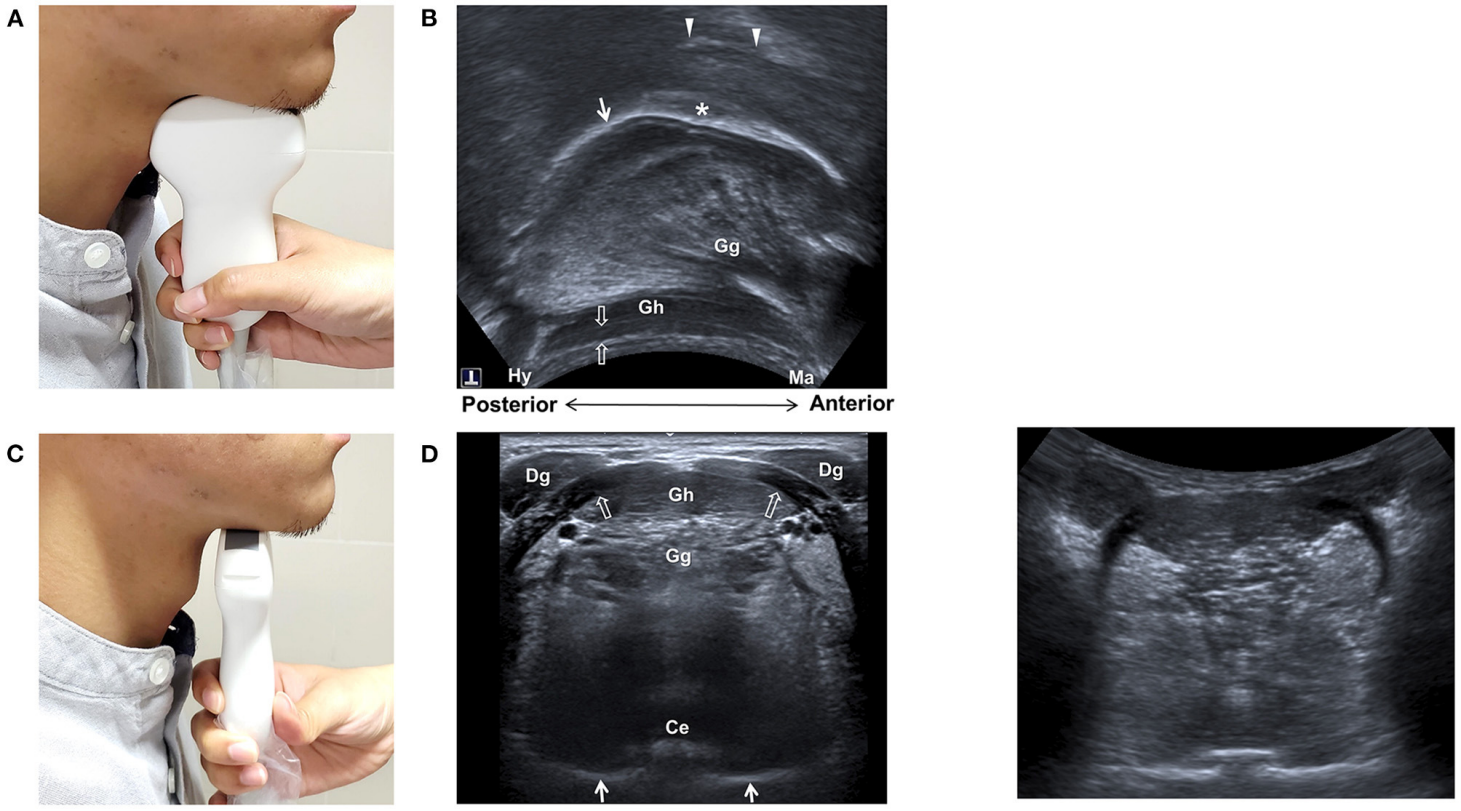
Ultrasonography of the tongue and mouth floor muscles. Positioning of the transducer for midsagittal view **(A)** and transverse view **(B)**, and sonographic imaging **(C,D)** of the tongue and mouth floor muscles. Ce, central groove of tongue; Dg, anterior belly of digastric muscle; Gg, genioglossus muscle; Gh, geniohyoid muscle; Hy, hyoid bone; Ma, mandible; arrow, tongue surface; arrow heads, palate; void arrows, mylohyoid muscle; asterisk, bolus.

Tongue movement is pivotal for the processing and propulsion of food in the oral preparatory and the oral phases of swallowing (34, 35). Observation of tongue movement is among the earliest and the most widely used application of US in the evaluation of swallowing function. The submental mid-sagittal view is the most commonly reported technique for observing tongue movement (Figure 1) (23, 24, 26, 36–38). The musculature of the tongue (genioglossus and intrinsic tongue muscle) and mouth floor (the anterior belly of digastric, geniohyoid, and mylohyoid muscles) can be depicted using either a sector or a curvilinear transducer. The sequential wavelike propagating movement of the tongue and bolus propulsion during swallowing can thus be assessed.

Ultrasonography inspection of the tongue was first described by Shawker et al. (23, 24), who also noticed that a patient with hypoglossal nerve palsy exhibited abnormal tongue movement during swallowing. Subsequently, a variety of methods have been used to analyze tongue movement, although many of these studies were preliminary and their clinical implications were limited [readers are referred to the comprehensive review by Stone for technical details (39)]. Previously reported techniques include using M-mode US to assess verticle movement of the tongue at a certain plane (37, 38, 40, 41), Doppler mode to analyze the hemodynamic changes of tongue vessels (42–44), measuring echogenicity of the tongue musculature (45), and temporal reconstruction of US imaging of the tongue surface (46). A descriptive scoring system for US observation of the oral stage of swallowing has also been described (47), based on tongue musculature, bolus control, initiation and coordination of tongue, and hyoid movement. In a more recent study, Ogawa et al. reported that patients with sarcopenic dysphagic had a reduced cross-sectional area and decreased echogenicity of the tongue when compared with the healthy elderly (48).

Ultrasonography can be used as a training tool in addition to a diagnostic one. Blyth et al. incorporated US imaging of the tongue as visual feedback in swallowing training of a patient with partial glossectomy and reported positive therapeutic effects in terms of skill acquisition and clinical aspiration signs (49).

It is worth noting that tongue movement is also critical for successful breastfeeding among infants. For example, infants with Down's syndrome often have reduced breastfeeding efficiency due to inadequate strength or coordination of tongue movement (50). In this regard, US, which does not cause discomfort and has no radiation, serves as a perfect tool for evaluating infant breastfeeding processes.

Undoubtedly, US could provide multifaceted and extensive information on the structure and movement pattern of the tongue. While previous studies have indicated certain useful parameters, an integration of these insights and a comprehensive evaluation of the complex movement of the tongue, as well as a prospective investigation of the diagnostic value of these US parameters, are required.

## Assessing Submental Muscles

Ultrasonography is an excellent tool for depicting muscular structures. Identification of submental muscles, namely, digastric, mylohyoid, geniohyoid, genioglossus muscles, and correlation with normal anatomy, has been described (15, 16, 18). These muscles are best outlined using a transverse submental US view (Figure 1). The reliability of US measurement of cross-sectional dimensions of the head and neck muscles, such as, the temporalis, masseter, digastric, and sternocleidomastoid muscles, has been reported (17). The test-retest reliability was found to be high for the anterior digastric, masseter, and sternocleidomastoid muscles while others showed moderate reliability. Furthermore, US measurement of the cross-sectional area of the anterior digastric muscle was found to be highly correlated to MRI findings (51).

The term “quantitative muscle ultrasound” was proposed by Ven Den Engel-Hoek et al. (45), who measured the muscle thickness and echo intensity of the tongue (by intraoral US) and the anterior belly of the digastric, mylohyoid, and geniohyoid muscles (transverse submental US view). Good measurement reproducibility was noted all muscles except for the mylohyoid muscle. Notably, an increased echogenicity of digastric and geniohyoid muscles and an increased thickness of tongue were found in five patients with Duchenne muscular dystrophy (DMD), compared with healthy subjects (19). US could thus be used as a quantitative tool for the evaluation of swallowing function in patients with neuromuscular diseases.

Ultrasonography may be beneficial in real-time observation of the extent of contraction of submental muscles. Similar to the technique used in observing tongue movement, Yabunaka et al. used submental sagittal M-mode US to evaluate verticle displacement and contraction duration of the geniohyoid muscle during swallowing and reported significant gender differences in different age groups (52). US imaging for real-time evaluation of genioglossus movement was also reported (53). Although high reproducibility was noted, the imaging analysis is quite complex and is mostly used for research purposes.

These results indicate that US evaluation of the structural integrity and contractile function of the submental muscles is feasible and reliable, opening up possibilities to more precise training or management. However, reference values of the cross-sectional diameter and echogenicity remain to be established. More research is required in order to provide clinical implications of these observations.

## Assessing Hyolaryngeal Movement

Excursion of the hyolaryngeal complex is one of the most important events in the pharyngeal phase of swallowing and is related to glottis closure and CPm relaxation (54–56). Over the past few decades, US has been increasingly applied to assess the pharyngeal phase of swallowing, owing to the advancement of its techniques and hardware. The most common application is the evaluation of hyoid bone displacement, (25–27, 29, 30, 57–59) and while observing hyoid-thyroid approximation (27, 28), lateral pharyngeal wall motion has also been reported (60).

**Figure 2:**
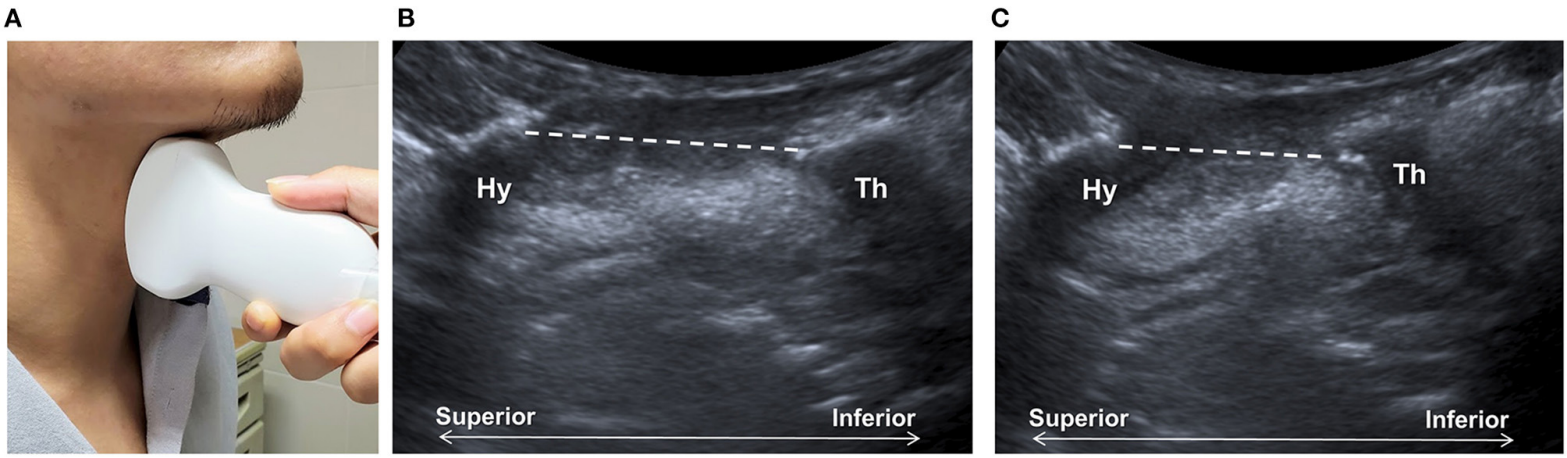
Ultrasonography assessment of thyroid-hyoid approximation. The transducer is placed at the midsagittal plane at the anterior neck **(A)**, between the thyroid cartilage (Th) and the hyoid bone (Hy). Sonographic imaging demonstrates the measurement of distance (dashed line) between the thyroid bone and thyroid cartilage at the resting position **(B)** and at maximal approximation **(C)**.

Shawker et al. (23, 24) used submental US to observe the tongue movement, raising the possibility of using this technique to observe the hyoid bone, although no reliable imaging could be obtained using a linear transducer. By placing a curvilinear transducer at the midline of the anterior neck, the approximation of hyoid bone and thyroid cartilage could be observed as a parameter to estimate larynx elevation (Figure 2) (27, 28). The measurement was found to be independent of gender and posture in another study (61). Huang et al. reported comparable results between measurements of US and VFSS and found a reduced hyoid-larynx approximation in the dysphagic stroke group (*N* = 40), compared with that in the non-dysphagic stroke group and the normal group (*N* = 15) (28). Similar findings were reported by Picelli et al. in a study of 19 patients with stroke. A correlation between the hyoid-larynx approximation and the Functional Oral Intake Scale (FOIS) was also identified (62). A recent study by Matsuo et al. reported an increased motion ratio of hyoid and thyroid displacement in patients with dysphagic stroke (*N* = 18) when compared with the healthy elderly (*N* = 18). Both sensitivity and specificity of detecting VFSS-verified dysphagia were found to be 88.9% using a motion ratio > 0.56 as the diagnostic criterion (63).

A variety of examination and data acquisition techniques have been reported for the measurement of hyoid bone displacement (25–27, 29, 30, 54–56). Most of the studies placed a curvilinear submental US transducer at the midsagittal plane, either hand-held or fixed by head frame with a transducer holder, to record hyoid bone movement during swallowing. These techniques had been successfully applied to investigate the effect of bolus volume and viscosity on the kinematics of the hyoid bone during swallowing. For example, thick liquid swallows resulted in the largest hyoid movement durations; and larger-volume swallows resulted in greater maximal hyoid bone displacement and forward peak velocity (64). Yabunaka et al. showed that the duration of hyoid bone movement increased with age while the maximal hyoid bone displacement decreased with age (30), indicating the possibility of using this parameter to diagnose dysphagia. In a recent study, the hyoid bone-laryngeal motion ratio (the hyoid bone displacement divided by the laryngeal displacement) was proposed as a parameter to evaluate hyolaryngeal excursion. However, there were no differences found between age and sex groups (65).

**Figure 3:**
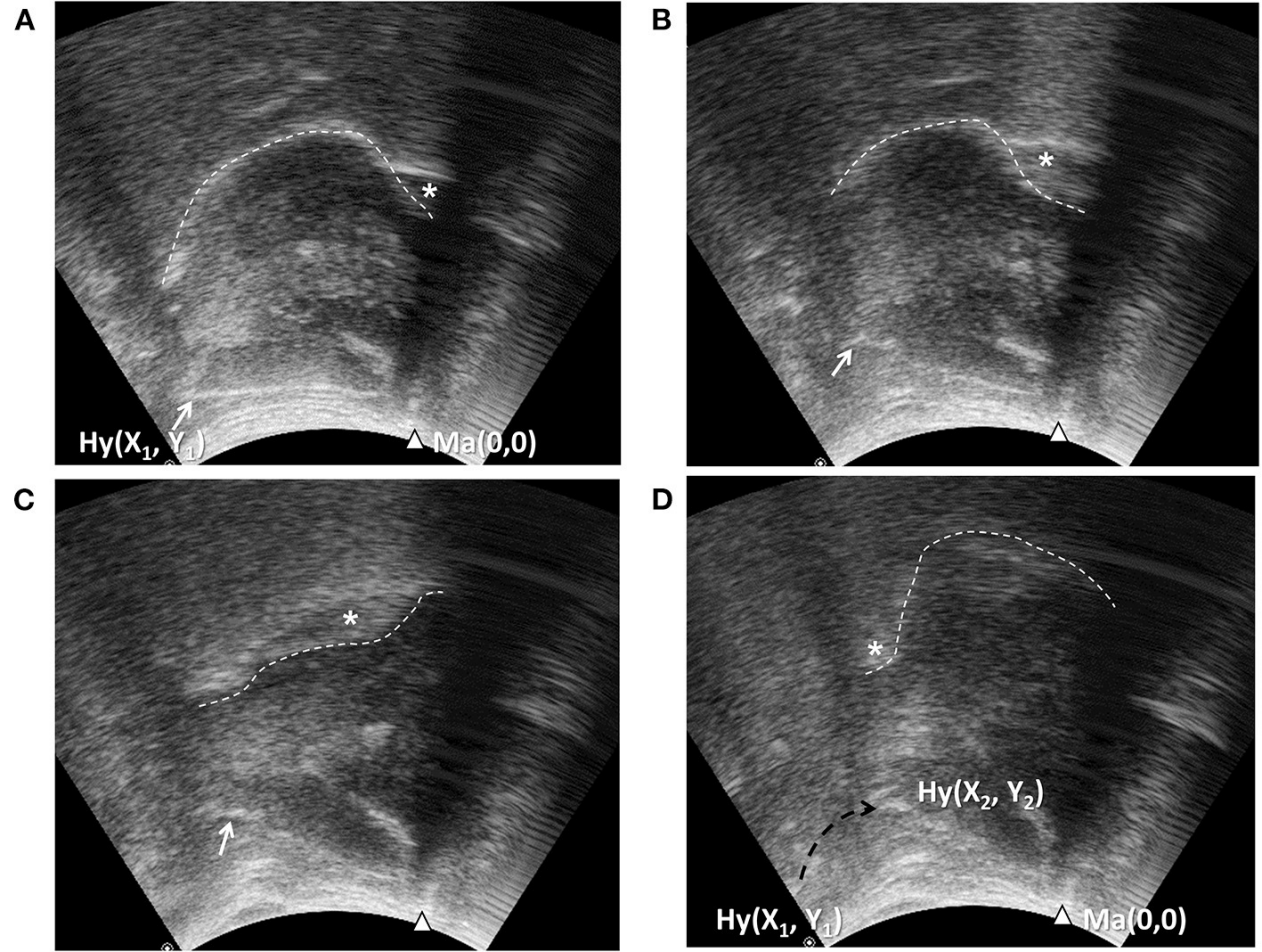
Ultrasonography assessment of tongue movement and hyoid bone displacement. Submental midsagittal ultrasonography (Figure 1A) showing the hyoid bone (arrow, Hy) at resting position **(A)**, during excursion **(B,C)**, and at maximal displacement **(D)**. The position of the mandible (arrow head, Ma) in each frame was used as the reference point to calculate the position coordinates of the hyoid bone. X_1_, Y_1_, position of the hyoid bone at resting position; X_2_, Y_2_, position of the hyoid bone at maximal displacement; dashed arrow: trajectory of the hyoid bone. Wave-like movement of tongue (dashed line) propagating the bolus backward could be observed simultaneously.

It is worth noting that most previous studies used the resting position of the hyoid bone as the reference point to calculate displacement. However, relative movement of the transducer and skin during the examination process can introduce a measurement error, if the transducer is positioned manually, for example, during bedside evaluation. Therefore, we propose using the mandible position as the reference point to calculate the positioning of the hyoid bone at rest and at maximal displacement (26) to account for the possible shifting of the transducer and the target during manual dynamic US examination. In addition, we used a broad submental US transducer that covers an area from the hyoid bone to the mandible, enabling visualization of the tongue and the hyoid bone simultaneously throughout the swallowing process (Figure 3). The reliability of hand-held submental US in measuring maximal hyoid displacement during swallowing showed high intra- and inter-rater agreement both in our result and a similar study (66).

In addition to modification of the technique, a cut-off value for diagnosing tube feeding dependent dysphagia in patients with stroke (*N* = 60) was proposed (a tongue thickness change of <1 cm and hyoid bone displacement of <1.5 cm, sensitivity 70–73%, and specificity 67%). The results by Lee et al. also supported this study, showing that a hyoid bone displacement of <1.35 cm detected penetration or aspiration with a sensitivity of 83.9% and a specificity of 81.0% (29).

**Figure 4:**
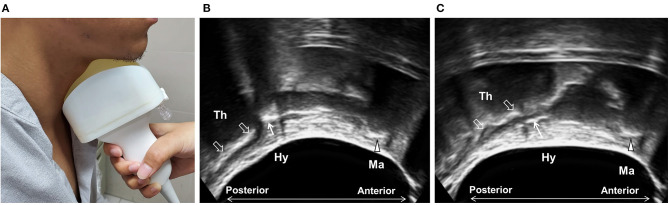
Ultrasonography using modified submental ultrasound transducer with water balloon. A water balloon was fixed in front of the transducer **(A)** to increase contact and to broaden the sonographic view, allowing for the simultaneous observation of the mandible (arrow head, Ma), the hyoid bone (arrow, Hy), and the thyroid cartilage (void arrows, Th) at rest **(B)** and during swallowing **(C)**.

One of the challenges of performing submental US is to maintain proper contact between the transducer and the submental area during the entire swallowing process. This can be further improved by setting a water balloon over the superior surface of the transducer (Figure 4) to substantially enhance imaging quality and allow for the examination of patients with prominent thyroid cartilage and reduced soft tissue at the neck. This also broadens the sonographic view and allows for the simultaneous observation of the thyroid cartilage, hyoid bone, and mandible, as well as other oral/lingual structures. It also makes the soft tissues and submental muscles easier to visualize. This modified technique showed high intra- and inter-rater reliability and a high correlation with VFSS with regard to the measurement of hyoid displacement (59). It is worth noting that this modification simplifies the examination technique and shortens the learning curve, thus facilitating the application of the technique by other medical personnel, potentially in long-term care facilities and home settings.

Despite the heterogeneous study designs and small case numbers in most previous studies, these results indicate the feasibility and reliability of US as a quantitative tool to evaluate hyolaryngeal excursion during swallowing. The accumulation of additional evidence can further establish the diagnostic value of US in pharyngeal phase dysphagia in different patient populations.

## Assessing Airway and Penetration-Aspiration

**Figure 5:**
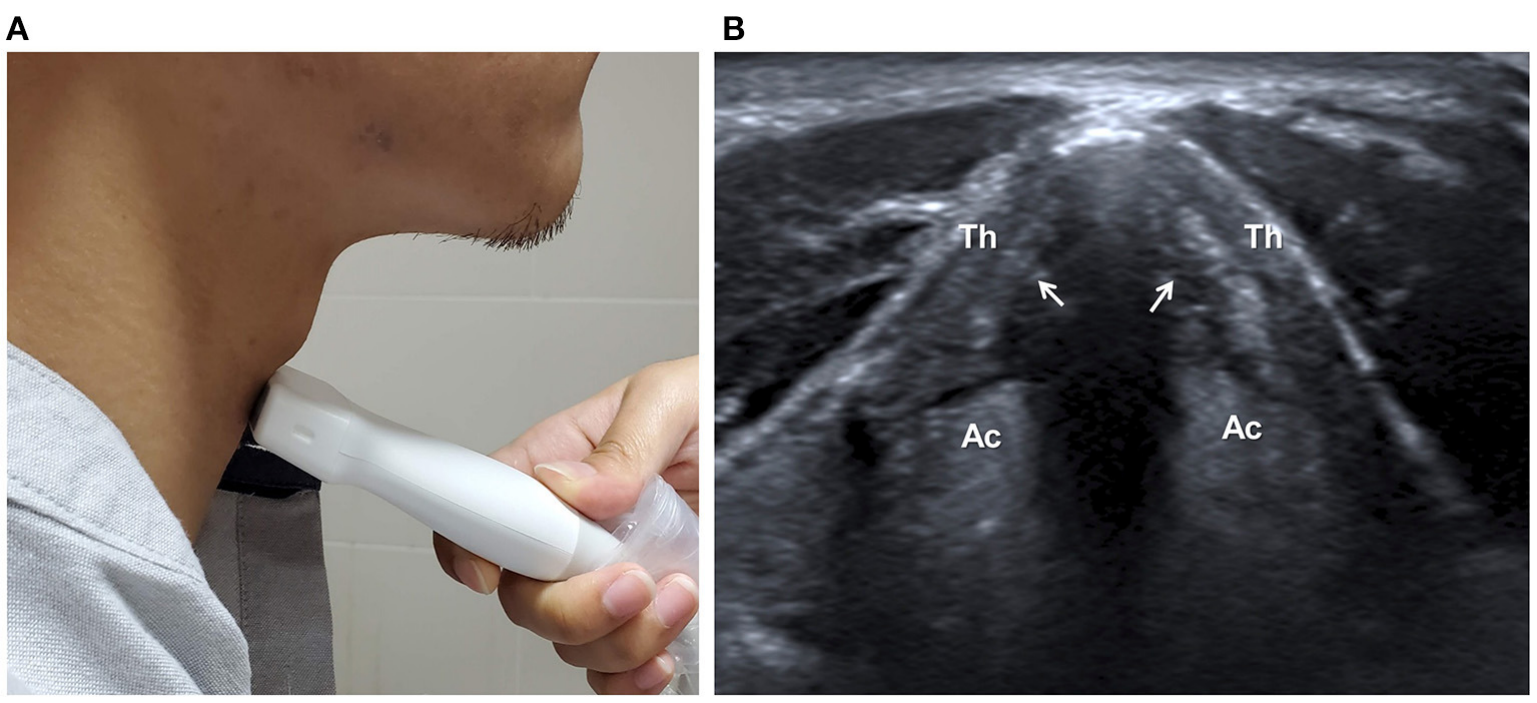
Ultrasonographic assessment of vocal cords. The transducer is placed anterior to the thyroid cartilage (Th) in transverse plane **(A)**. The structure and movement of vocal cords (arrows) can be observed **(B)**. Ac, Arytenoid cartilage.

Adequate vocal fold function is critical to airway protection during swallowing. The structure and movement of vocal folds could be directly observed by B-mode ultrasound (Figure 5). A prospective study reported repeatable results of US observation of airway structures, such as, the tongue, the hyoid bone, the thyrohyoid membrane, the epiglottis, the thyroid cartilage, the vocal cords, the cricoid cartilage, the cricothyroid membrane, and the trachea (22). A multi-institutional observational study indicated the high sensitivity of US to detect vocal cord paralysis, although the visibility ranged from 41 to 86% (lower in male than in female) and was highly operator-dependent (67).

Due to the hindering of the thyroid cartilage and excursion of the hyolaryngeal complex during swallowing, direct observation of aspiration by US is much more challenging. Miura et al. were the first to propose the feasibility of observing aspirated bolus in the trachea in the sagittal plane (21). The sensitivity of aspiration detection was 0.64, and the specificity was 0.84, using VFSS as standard. The same group later reported the use of US to detect pyriform sinus residue (sensitivity 92.0% and specificity 71.9%) and epiglottic vallecular residue (sensitivity 86.7% and specificity 63.6%) using the FESS result as reference (68).

While more evidence is needed, US could serve as a quick screening tool for vocal cord paralysis and post-swallowing residual, providing indirect evidence of pharyngeal dysphagia.

## Guiding Intervention of Upper Esophageal Sphincter Dysfunction

The upper esophageal sphincter (UES) comprises the inferior pharyngeal constrictor, the CPm, and the cervical esophagus, with CPm being the primary component of sphincter function. The opening of the UES marks the last vital step of the pharyngeal stage and the beginning of the esophageal stage. UES dysfunction, often due to inadequate relaxation of the CPm, may result in excessive pharyngeal residual and post-swallowing aspiration.

**Figure 6:**
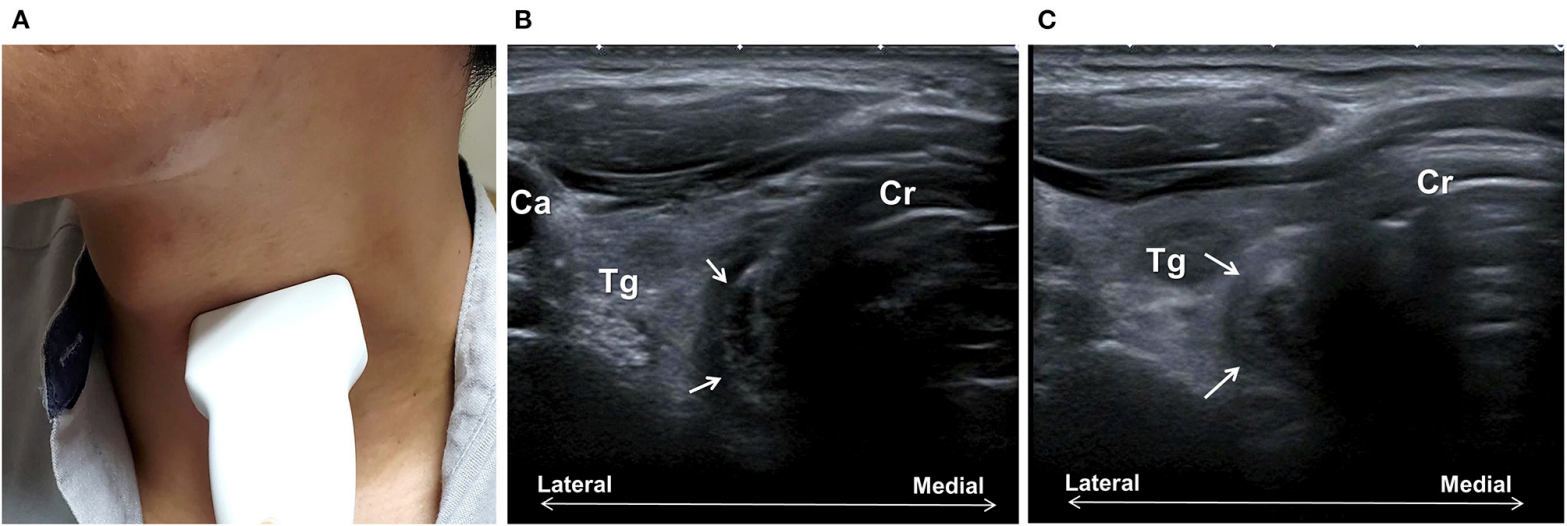
Ultrasonography assessment of the cricopharyngeal muscle (CPm). The transducer is placed transversely at the left of the trachea at the cricoid cartilage (Cr) level **(A)**. The CPm (arrows) appears as an oval-shaped multiplayer tubular structure **(B)**. Bolus passing can be observed with the rounding of the CPm during swallowing **(C)**. Ca, common carotid artery; Tg, thyroid gland.

Direct visualization of the CPm is possible with the conventional US. When a linear transducer is placed at the left of the anterior neck at the level of the cricoid cartilage, CPm could be seen as a multilayer oval structure (Figure 6). The normal diameter of the closed and open UES has been reported (31). Although the relaxation parameter in patients with dysphagia is currently lacking, it indicates the feasibility of using US to observe structural changes of the CPm during swallowing.

Botulinum toxin injection of CPm has been applied in the treatment of UES dysfunction since 1995 (69). Most studies reported a high percentage of positive outcomes (>75%) and low adverse effects. While most previous procedures were conducted by endoscopy under anesthesia, CT-guided, and electromyographic-guided (69–71), US-guided injection has recently attracted increasing attention. Concurrent electromyography could be used to confirm the localization of the needle before injection. Wang et al. described the procedure to localize the CPm by US in both transverse and longitudinal views and reported that dysphagic cases were successfully treated by US-guided botulinum toxin injection of CPm (32, 33). A recent single-arm prospective study by Xie et al. injected CPm botulinum toxin guided by US, catheter balloon, and electromyography in patients with neurogenic UES dysfunction (*N* = 21) (72). Post-injection evaluation showed improved swallowing function in terms of FOIS, UES opening parameters, penetration-aspiration scale, and decreased pharyngeal residue. Due to its non-invasive and radiation-free nature, US has shown high potential as a clinically useful tool for evaluation of the UES structure and for guided intervention in the UES dysfunction. Further large-scale trials are warranted to confirm its safety and efficacy in comparison to conventional techniques.

## Conclusion and Perspectives

The past few decades has seen increased research attention on the versatility of US in the evaluation of swallowing function. The most common applications are assessing tongue and hyolaryngeal movement. While techniques for assessing tongue and other swallowing-related muscle structures are fairly well-described, dynamic evaluation of these muscle movements during swallowing remains to be refined. Further research is suggested to elucidate the effect of altered submental muscle contraction on the efficiency and safety of swallowing, particularly, in the diseased population. In future, comprehensive image analysis may provide further insights into the diagnostic value of the complex movement pattern of tongue/submental muscles.

With regard to the evaluation of hyolaryngeal excursion, US provides a simple and reliable method for quantitative measurement. Accumulating evidence indicates that decreased hyoid bone displacement or hyoid-thyroid approximation is associated with severity of dysphagia or the risk of penetration/aspiration. These parameters can be used for bedside screening of dysphagia. The predicting value of hyoid bone displacement on functional outcome, e.g., aspiration pneumonia, warrants further investigation.

Numerous applications of US are emerging in the diagnosis and management of dysphagia, for example, assessing pharyngeal residue, assessing aspiration and UES function, and guided intervention for UES dysfunction. Nevertheless, more clinical studies are needed to establish the diagnostic criteria of US evaluation of UES dysfunction. In addition, large-scale randomized control trials are needed to confirm the benefit of US-guided CPm injection of botulinum toxins in patients with dysphagia and to compare the safety and efficacy of US guidance with other methods.

Non-invasive, radiation-free, simple to use, and portable US is an ideal tool as a first-line screening for swallowing function, particularly, in facilities where formal instrumental examinations or physicians are not available.

## Author Contributions

M-YH designed the research, performed the literature review, and wrote the manuscript. C-HW performed the literature review and revised the manuscript. T-GW supervised the research and revised the manuscript. All authors contributed to the article and approved the submitted version.

## Conflict of Interest

The authors declare that the research was conducted in the absence of any commercial or financial relationships that could be construed as a potential conflict of interest.

## Publisher's Note

All claims expressed in this article are solely those of the authors and do not necessarily represent those of their affiliated organizations, or those of the publisher, the editors and the reviewers. Any product that may be evaluated in this article, or claim that may be made by its manufacturer, is not guaranteed or endorsed by the publisher.
